# Self-Efficacy is Associated with Health Behaviors Related to Obesity and Cardiovascular Risk among Hispanic/Latinx and Somali Immigrants to the United States

**DOI:** 10.21203/rs.3.rs-6001516/v1

**Published:** 2025-02-19

**Authors:** Brianna Tranby, Irene Sia, Matthew Clark, Paul Novotny, Abby Lohr, Laura Suarez Pardo, Christi Patten, Sheila Iteghete, Katherine Zeratsky, Thomas Rieck, Luz Molina, Graciela Porraz Capetillo, Yahye Ahmed, Hana Dirie, Mark Wieland

**Affiliations:** Mayo Clinic; Rochester Healthy Community Partnership; Mayo Clinic; Mayo Clinic; Mayo Clinic; Mayo Clinic; Mayo Clinic; Mayo Clinic; Mayo Clinic; Mayo Clinic; Rochester Healthy Community Partnership; Rochester Healthy Community Partnership; Rochester Healthy Community Partnership; Rochester Healthy Community Partnership; Mayo Clinic

**Keywords:** Health behaviors, healthy eating, physical activity, immigrant communities, cardiovascular risk, self-efficacy

## Abstract

**Background:**

Self-efficacy theory proposes that confidence to engage in a health behavior is associated with engaging in that specific behavior. Most research examining self-efficacy has been conducted with white young adult populations. This secondary analysis examined the association of self-efficacy (i.e., confidence) for healthy eating and physical activity in two immigrant communities.

**Methods:**

At enrollment into the Healthy Immigrant Community study, a clinical weight management and cardiovascular risk reduction intervention set in southeastern [state], 475 participants completed assessments about their confidence for healthy eating and physical activity. Measurements also included self-reports of dietary quality and intake, physical activity, quality of life, and biometric assessments. Study materials were available in English, Spanish, and Somali.

**Results:**

In total, 450 adults (Hispanic/Latinx = 267; Somali = 183) completed measures at baseline and were included for analysis. Their average age was 45 years (range 18–87) and 59% were female. Confidence for healthy eating was significantly associated with self-report of eating healthy snacks (*p* = < 0.0001) and less consumption of high-calorie drinks (*p* = 0.02) and regular soda (*p* = < 0.0001). Confidence to be physically active was significantly associated with more self-reported physical activity (*p* = < 0.01).

**Conclusions:**

Confidence to eat healthy and be physically active appears to be associated with having a healthier diet and higher levels of physical activity. Given the large sample size and strength of the associations, it also appears that the theoretical model of self-efficacy can be effectively measured and applied within these immigrant populations. Self-efficacy theory may be useful in understanding potential mediating mechanisms when designing future interventions with immigrant communities.

**ClinicalTrials.gov registration:**

NCT05136339; April 23, 2022

## Introduction

Identifying mediating mechanisms that facilitate an individual’s ability to improve their health behaviors can be useful in designing and delivering health behavior interventions. One possible mediating mechanism that is a component of social cognitive theory is self-efficacy, which theorizes that an individual’s confidence to engage in a behavior is predictive of the individual engaging in that specific behavior.[1] Self-efficacy for eating has been shown to improve after participation in behavioral weight management programs,[2] and in a sample of 1,740 patients seeking bariatric surgery, lower eating self-efficacy was associated with higher rates of binge eating episodes, food addiction scores, night eating syndrome, and negative mood.[3]

While low self-efficacy for eating is associated with higher levels of anxiety[4] and lower rates of having bariatric surgery,[5] improvements in self-efficacy after weight loss surgery are predictive of long-term weight loss maintenance.[6] In a sample of 676 worksite “wellness champions,” high stress level was associated with low self-efficacy for following a healthy diet over a five-year period.[7] In a sample of over 13,000 members of a wellness center, high stress was associated with low-self efficacy for being physically active.[8]

How self-efficacy theory and measures apply to immigrant populations is a growing area of investigation. A recent literature review called for more research that examines psychosocial factors that impact physical activity level in Black and Latina individuals.[9] A physical activity intervention based on social cognitive theory demonstrated effectiveness in a sample of Spanish-speaking Latinas,[10] as did a physical activity pilot project with Somali women.[11] Our team previously reported that negative mood was associated with lower confidence for eating healthy among 449 Hispanic and Somali immigrants, but found no association between negative mood and confidence to be physically active.[xx] A review of four community health worker-led interventions with Asian immigrants found that self-efficacy and physical activity improved significantly among participants in the treatment groups.[13] A combined analysis of 176 Latina adults who participated in one of two exercise interventions found higher self-efficacy two months into the program was associated with less relapse.[14] Additionally, food insecurity was also found to be associated with low self-efficacy and heritage language proficiency among immigrants to the United States.[15]

The current study is embedded within the Healthy Immigrant Community (HIC) study, which is a cluster randomized trial to assess the effectiveness of a social network-informed, community-based participatory research (CBPR) derived health promotion intervention on measures of obesity and cardiovascular risk among Hispanic and Somali immigrant populations.[xx] HIC is a product of [city] Healthy Community Partnership (RHCP), which is a 20-year CBPR partnership in southeast [state] that collaborates with immigrant and refugee populations to co-create interventions that promote health equity for community health priorities.[xx] RHCP is productive and experienced at using a CBPR approach to co-develop and test community-based interventions to reduce the accumulation of cardiovascular risk after immigration.[xx]

This study is a cross-sectional analysis of baseline data from the RHCP HIC study. Our hypothesis was that the association between self-efficacy and healthy eating and physical activity behaviors previously described among predominantly white, affluent study populations[19] would also be found among immigrant communities in the United States.

## Methods

### Setting and Participants

The [institution name] Institutional Review Board approved the HIC study (NCT0513633) in 2021. All participants provided written informed consent. The study setting and participants were described in previous papers.[xx, xx] Briefly, HIC is set is Olmsted County in southeastern [state] (2021 estimated population: 163,436).[20]

Approximately 11% of the population were born outside of the US, of which 55% have become US citizens,[20] and 14% of people living in the county speak a language other than English at home.[21]

The study utilized baseline data from the RHCP HIC study. Through this CBPR-informed study, 52 members of the immigrant communities, identified by RHCP community partners, were trained as health promoters (HP) and delivered the 12-month intervention to 472 participants in their existing social networks either immediately following baseline measures or after a one-year delay (delayed-intervention control group). Baseline data was collected in June-August 2022

HIC inclusion criteria included age ≥ 18 years, self-reported Hispanic/Latinx or Somali ethnicity, being a member of a HP’s social network, and being willing to participate in all study procedures. Participants were excluded if they were pregnant at the time of enrollment or had a medical condition or disability that prevented them from being more physically active. Baseline measures obtained by study staff and community volunteers included: 1) biometric measurements such as height, weight, blood pressure, glucose; 2) survey measures assessing dietary quality and intake, physical activity, and quality of life; and 3) demographics. Assessments were carefully translated and available in English, Spanish, or Somali.[22]

### Primary Outcome Measures

Self-Efficacy: As previously described, participants completed the adapted Patient Centered Assessment and Counseling for Exercise plus Nutrition (PACE+) survey at baseline.[23] Two items assessed self-efficacy: “How confident are you that you can eat a healthy diet?” and “How confident are you that you can participate in regular exercise or physical activity?” Both items were rated as a percentage interval (0%=not at all confident; 25%; 50%=somewhat confident; 75%; 100%=very confident).

Nutritional Behaviors: The Food Behavior Checklist, which is validated for use among diverse communities, was used to assess dietary quality and intake.[24, 25] Questions included in this analysis were: “Do you eat fruits or vegetables as snacks?”, “Do you drink fruit drinks, punch, or sports drinks?, and “Do you drink regular soda?”. The items were rated on a 4-point Likert scale (no; yes, sometimes; yes, often; yes, everyday), but “often” and “everyday” were combined on analysis to produce three variables (no; sometimes; often).

Participants also completed the Automated Self-Administered 24-hour (ASA24^®^) Dietary Assessment Tool[26] on a computer with assistance from study staff and an interpreter if needed. The Healthy Eating Index (HEI) is a score produced by the ASA24^®^ based on 10 recommended dietary components including high intake of fruit, vegetables, protein, dairy, whole grains, and low intake of fats, cholesterol, and sodium. Each component is weighted with a possible score from 0–10 with a maximum overall score out of 100; higher scores indicate healthier diets.

### Physical Activity

The International Physical Activity Questionnaire (IPAQ) short form was used to measure physical activity levels in the baseline survey.[27] Physical activity was defined as “any activity that increases your heart rate and makes you breathe harder some of the time” including at work, house and yard work, and sports or exercise. “Vigorous” activity was defined as one that “takes much more physical effort and makes you breathe a lot harder than normal” like “heavy lifting, digging, aerobics, or fast bicycling.” “Moderate” activity was one that “takes somewhat more physical effort and makes you breathe a little harder than normal” like “carrying light loads or bicycling at a regular pace.” Participants were instructed to not include walking as “moderate” physical activity.

Participants reported separately the total number of days out of the last seven days that they did “vigorous” and “moderate” activities for at least 10 minutes at a time. They also answered the question “On the days you did do [vigorous or moderate] physical activity, how many minutes total did you usually spend on one of those days?” Participants reported how many days out of the last seven days that they walked for at least 10 minutes at a time, including “all types of walking at work and at home” and the total minutes they usually spent walking on one of those days. Lastly, participants were asked about time spent sitting “on weekdays while at work, at home, or any other place.” The total time usually spent sitting on a weekday during the last seven days was reported in hours and minutes.

The recommended scoring criteria for the IPAQ contains three categories: Inactive, Minimally Active, and Health-Enhancing Physical Activity (HEPA) Active. The “minimally active” category is defined as “≥3 days of vigorous activity at least 20 minutes/day, ≥ 5 days of moderate-intensity activity or walking at least 30 minutes/day, or ≥ 5 days of activity achieving a minimum of least 600 MET-min (multiples of the resting metabolic rate)/week.” As in our previous analysis,[xx] the “minimally active” and “HEPA active” categories were combined because the criterion for both exceed public health recommendations for physical activity; thus, two variables were analyzed (active, inactive).

### Data Analysis

**Figure 1:**
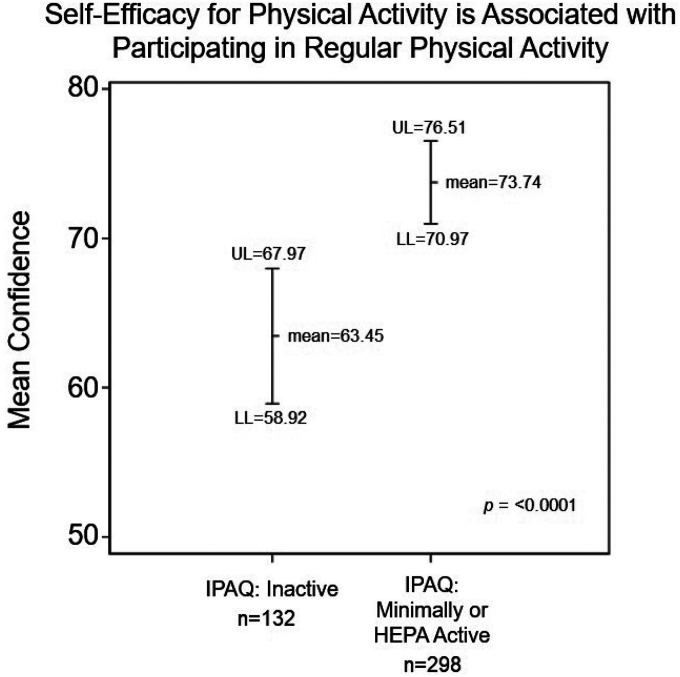
Association between confidence to participate in regular exercise or physical activity Mean and 95% confidence intervals based on t-test treating confidence as a continuous variable. LL=Lower Limit (95% confidence limit) for mean; UL=Upper Limit (95% confidence limit) for mean.

Kruskal-Wallis tests were used to analyze continuous variables by categorical variables such as race, confidence levels, and IPAQ categories. Analyses were two-sided using 5% type I error rates. Associations between continuous variables were assessed using Spearman correlation coefficients and a t-test was performed for Fig. 1. Analyses were performed using SAS version 15.1 (SAS Institute Inc. Cary, NC, USA).

## Results

In total, 450 participants who identified as Hispanic/Latinx (n = 267, 59%) or Somali (n = 183, 41%) and completed baseline measures were included in this analysis. Of the 450 participants, 261 (59%) were female, the mean age was 45 years (range = 18–87; SD = 14.3), and 167 (37%) had no health insurance plan in the past 12 months. Although missing in 89 (20%) participants, only 38 (11%) reported English as the language they most commonly speak at home, and 248 (55%) reported speaking English “not at all” or “not very well” compared to those who spoke “well” or “very well” (Table 1).

For the item “How confident are you that you can eat a healthy diet?”, 6 (1%) reported being “0% Not at all Confident”, 27 (6%) were “25%” confident, 131 (29%) were “50% Somewhat confident”, 119 (26%) were “75%” confident, and 167 (37%) were “100% Very Confident”. For the item “How confident are you that you can participate in regular exercise or physical activity?” 17 (4%) were “0% Not at all Confident”, 34 (8%) were “25%” confident, 133 (30%) were “50% Somewhat confident”, 116 (26%) were “75%” confident, and 150 (33%) were “100% Very Confident”.

Overall, 407 (90%) reported eating fruits or vegetables as snacks either “sometimes”, “often”, or “every day”. Additionally, 277 (62%) reported drinking fruit drinks, punch, or sports drinks, and 273 (61%) reported drinking regular soda either “sometimes”, “often”, or “every day”. There was no significant difference between Hispanic/Latinx and Somali participants in drinking fruit drinks, punch, or sports drinks (*p* = 0.4). Hispanic/Latinx participants were more significantly more likely to report drinking regular soda “sometimes”, “often”, or “every day” than Somali participants (*p* = < 0.001), and to report eating fruits and vegetables as snacks “every day” (*p* = 0.03).

Among the 430 participants who completed the IPAQ, 298 (69%) met criteria as “minimally or HEPA active”, the mean total MET minutes per week was 3,913 (range = 0–40,320; SD = 6,491), and the mean sitting minutes per week was 314 (range = 0–1,300; SD = 277).

### Confidence and Nutrition

Participants who reported being more confident in eating a healthy diet were more likely to report eating fruits and vegetables as snacks (*p* = < 0.001) (Table 2). No participants who were “not at all confident” reported eating fruits or vegetables as snacks, while 60% of those who were “very confident” ate fruits and vegetables every day.

Respondents who were more confident in healthy eating were also less likely to report drinking fruit drinks, punch, or sports drinks (*p* = 0.018). Only 3% of respondents who were “not at all confident” reported drinking not drinking fruit drinks, punch, or sports drinks, while 44% of those who were “very confident” reported not drinking them.

Additionally, participants who reported being more confident in eating healthy were less likely to drink regular soda (*p* = < 0.001). Only 1% of respondents who were “not at all confident” reported not drinking regular soda, while 47% of those who were “very confident” reported not drinking them.

Confidence to eat healthy was not associated with gender, income, or education in our multivariate analysis. Confidence to eat healthy was only marginally associated with ASA24 (Spearman correlation = 0.14; *p* = 0.0026) and was not associated with the number of servings of fruits or vegetables. All three confidence associations were statistically significant among Hispanic/Latinx participants (*p* = < 0.0001 for eating fruits/vegetables as snacks; *p* = 0.03 for drinking fruit drinks/punch/sports drinks; *p* = 0.02 for drinking regular soda). Among Somali participants, the first two variables approached significance (*p* = 0.06 for eating fruits/vegetables as snacks; *p* = 0.06 for drinking fruit drinks/punch/sports drinks) but was significant for drinking regular soda (*p* = < 0.0001).

### Confidence and Physical Activity Level

Confidence to participate in regular exercise or physical activity was significantly associated with IPAQ categories (*p* = 0.0013). Sixty-three percent of those who were “not at all confident” were classified as “IPAQ: Inactive” compared to only 22% who were “very confident” (*p* = 0.001). Among those classified as “Minimally or HEPA Active,” 78% were “very confident” compared to only 38% who were “not at all confident”. Confidence to be physically active also had moderate Spearman correlations with IPAQ walking (Spearman = 0.25; *p* = < 0.0001), moderate (Spearman = 0.21; *p* = < 0.0001), vigorous (Spearman = 0.17; *p* = 0.0004), and total (Spearman = 0.20; *p* = < 0.0001) MET-Minutes per week. There was no correlation between confidence and mean minutes sitting per week (Spearman = 0.006; *p* = 0.93).

When confidence was treated as a continuous variable instead of categorical, the mean for participants classified as “IPAQ: Inactive” was 63.45 (95% CI = 58.92–67.97) compared to a mean of 73.74 for those who were “IPAQ: Minimally or HEPA Active” (95% CI = 70.97–76.51; *p* = < 0.0001) (Fig. 1). The association between confidence and IPAQ category was also statistically significant among both Hispanic/Latinx (*p* = 0.03) and Somali respondents (*p* = 0.02) with those who were “very confident” more likely to be classified as “Minimally or HEPA Active”.

## Discussion

Self-efficacy theory postulates that an individual’s confidence to successfully perform a behavior is predictive of the individual engaging in that behavior. Numerous studies have reported an association between self-efficacy and health behaviors.[2–8] Previous studies with smaller sample sizes reported an association between self-efficacy for healthy eating and dietary self-report, as well as self-efficacy for physical activity and self-reported physical activity among Latina, Somali, and Asian Americans groups.[9–11, 13–15] This analysis extends those findings to a larger population of adult immigrants in the United States. Thus, including strategies for increasing self-efficacy for healthy eating and physical activity in culturally tailored weight management interventions may help improve the effectiveness of these cardiovascular risk reduction programs with immigrant populations.

In terms of the nutritional findings, the relationship between self-efficacy and nutritional intake has been well documented.[2, 4, 6] This report extends previous findings to a larger sample of immigrants to the United States. The finding that self-efficacy for physical activity was associated with self-report of physical activity level has also been previously reported.[7–11, 28, 29] Given that confidence for being physically active is associated with actual reported physical activity, interventions designed to promote physical activity in immigrant populations should include strategies for increasing self-efficacy for being physically active.

When translating theoretical concepts and measures into other languages and assessing their usefulness for clinical interventions with other cultures, it is important to examine not only the association of the translated measures with other validated measures, but also the range of scores on the measures.[22] For example, if participants are clustered to one response option on an item, it may indicate that the concept may not apply to that understudied population, or that the translation was done poorly. In this project, careful steps were taken to translate the items, and there was a wide range of responses in line with previous findings. This suggests that the concepts were applicable to these populations and the items were successfully translated.

This project has several limitations to note. First, nutritional intake was not directly measured or recorded. More direct measurement of nutritional intake would strengthen the project. Physical activity level was also self-reported, and direct measurement of physical activity level would improve the study. Additionally, this is a baseline report, so how these variables may change secondary to participation in a cardiovascular risk reduction intervention has yet to be assessed. Future research should consider examining changes in self-efficacy levels before and after behavioral interventions and their ongoing association over time.

In conclusion, in this large sample of immigrants to the United States, self-efficacy for healthy nutrition was associated with consuming health snacks, lower intake of high calorie beverages and drinking less soda. Self-efficacy for physical activity level was also associated with a higher level of self-reported physical activity. Investigators and clinicians should consider incorporating strategies for improving self-efficacy into weight management interventions that are culturally tailored and co-developed with immigrant communities.

## Figures and Tables

**Table 1:** Participant demographics at baseline

	Total (n = 450)
**Age**	
Mean (SD)	44.7 (14.3)
Range	(18–87)
**Gender**	
Missing	5
Male	169 (38%)
Female	261 (59%)
Other	15 (3%)
**Ethnicity**	
Hispanic or Latinx	267 (59%)
Somali	183 (41%)
**Education**	
Missing	3
Some high school or less	184 (41%)
High School graduate or GED	119 (27%)
Some college or technical school	85 (19%)
4-year degree or beyond	59 (13%)
**Income**	
Missing	25
$0 to $29,999	228 (54%)
$30,000 to $49,999	111 (26%)
$50,000 or more	86 (20%)
Have you had a health insurance plan in the past 12 months?	5
Missing	278 (63%)
Yes	167 (37%)
No	
**What language do you most commonly speak at home?**	89
Missing	38 (11%)
English	121 (34%)
Somali	197 (55%)
Spanish	5 (1%)
Other	
**How well do you speak English?**	
Missing	7
Not at all	92 (21%)
Not very well	156 (35%)
Well	97 (22%)
Very well	98 (22%)

**Table 2:** Associations between confidence to eat healthy and other nutritional endpoints at baseline

	0% Not at All Confident	25%	50% Somewhat Confident	75%	100% Very Confident	Total	*p* value
	(n = 6)	(n = 27)	(n = 131)	(n = 119)	(n = 167)	(n = 450)	
**Do you eat fruits or vegetables as snacks?**							
Missing	0	0	0	4	5	9	**< 0.0001** *
No	0 (0%)	6 (22%)	9 (7%)	9 (8%)	10 (6%)	34 (8%)	
Yes, sometimes	5 (83%)	18 (67%)	93 (71%)	63 (55%)	80 (49%)	259 (59%)	
Yes, often	1 (17%)	1 (4%)	17 (13%)	30 (26%)	31 (19%)	80 (18%)	
Yes, everyday	0 (0%)	2 (7%)	12 (9%)	13 (11%)	41 (25%)	68 (15%)	
**Do you drink fruit drinks, punch, or sports drinks?**							
Missing	0	0	0	4	3	7	**0.0179** *
No	5 (83%)	6 (22%)	38 (29%)	44 (38%)	73 (45%)	166 (38%)	
Yes, sometimes	0 (0%)	16 (59%)	79 (60%)	61 (53%)	70 (43%)	226 (51%)	
Yes, often	0 (0%)	3 (11%)	11 (8%)	7 (6%)	15 (9%)	36 (8%)	
Yes, everyday	1 (17%)	2 (7%)	3 (2%)	3 (3%)	6 (4%)	15 (3%)	
**Do you drink regular soda?**							
Missing	0	1	1	1	0	3	**< 0.0001** *
No	2 (33.%)	1 (4%)	39 (30%)	50 (42%)	82 (49%)	174 (39%)	
Yes, sometimes	1 (17%)	16 (62%)	63 (49%)	53 (45%)	71 (43%)	204 (46%)	
Yes, often	2 (33%)	4 (15%)	21 (16%)	12 (10%)	10 (6%)	49 (11%)	
Yes, everyday	1 (17%)	5 (19%)	7 (5%)	3 (3%)	4 (2%)	20 (5%)	

* Kruskal-Wallis test

## Data Availability

The data that support the findings of this study are available from the corresponding author, BNT, upon reasonable request.
